# All-optical tunable slow-light based on an analogue of electromagnetically induced transparency in a hybrid metamaterial

**DOI:** 10.1039/d1na00232e

**Published:** 2021-08-13

**Authors:** Chengju Ma, Yuebin Zhang, Yao Zhang, Shiqian Bao, Jiasheng Jin, Mi Li, Dongming Li, Yinggang Liu, Yiping Xu

**Affiliations:** School of Science, Xi'an Shiyou University Xi'an 710065 P. R. China chengjuma@xsyu.edu.cn; School of Physics and Optoelectronic Engineering, Yangtze University Jingzhou 434023 P. R. China

## Abstract

We demonstrate and analyze the use of metamaterials featuring an analogue of electromagnetically induced transparency (EIT) in slow light technology. For most metamaterials, EIT-like effects suffer from intrinsic ohmic loss, and the metamaterial-based slow-light effect can only be tuned passively, which limits their application in slow light devices. We propose a hybrid metamaterial with a unit cell composed of a ring resonator formed from photoactive silicon (Si) and a rectangular bar formed from metallic silver (Ag). Based on an analogue of EIT in the designed hybrid metamaterial, we theoretically demonstrate an all-optical tunable slow-light effect in the telecommunication window. We successfully demonstrate the possibility of designing novel all-optical tunable chip-scale slow-light devices that could be used in optical buffering.

## Abstract 2

Based on an analogue of EIT in a proposed hybrid metamaterial, we theoretically demonstrate an all-optical tunable slow-light effect, and show the possibility of designing novel all-optical tunable chip-scale slow-light devices.

## Graphical abstract

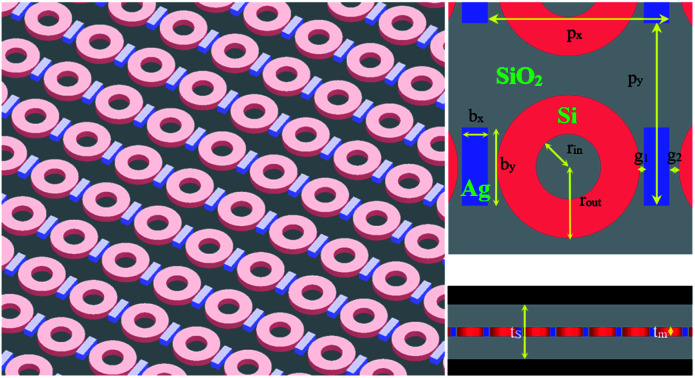

## 1. Introduction

Slow light means making an optical signal slow down to thus be able to control it. Slow light is a perennial topic of interest for researchers, who seek to utilize its full potential in optical buffers, switching, memory and quantum optics, in which full control of the speed of light is necessary.^1^ So far, most of the approaches to obtain a slow-light effect rely on strong material dispersion based on nonlinear optical processes, such as electromagnetically induced transparency (EIT),^2^ stimulated Brillouin scattering (SBS),^3^ coherent population oscillations (CPO),^4^ stimulated Raman scattering (SRS),^5^ and optical parametric amplification (OPA).^6^ The group velocity of light can also be significantly reduced by designing periodic microstructures, such as those in photonic crystals (PHCS),^7^ Fabry–Perot (F–P) resonators,^8^ surface plasmon polaritons (SPPs),^9^ and fiber Bragg gratings (FBG).^10^ Although a series of methods to demonstrate the slow-light effect have been proposed, to date, most of them have exhibited inherent limitations that hinder their practical application. For instance, EIT requires stable gas lasers and a low-temperature environment. CPOs and SBS are very narrowband, owing to the narrow transparency window of CPOs and the narrow Brillouin gain bandwidth. SPPs are very sensitive to surface roughness and relatively difficult to excite. PHCs are normally highly multimodal.^11^ However, slow-light elements with characteristics such as integration, broadband, active control, low loss, and high fidelity, are urgently needed for all optical networks (AON), which has driven great efforts to exploit new methods or advanced materials to achieve a slow-light effect.

Metamaterials are generally structured with a patterned metallic subwavelength unit cell to yield specific electromagnetic properties. These artificial materials, in which electromagnetic waves propagate in a nonconventional way, provide a unique possibility to design novel types of slow-light devices.^12^ Recently, the achievement of EIT in metamaterials, which is also called an EIT analogue or EIT-like effect, has opened new avenues for realizing more efficient slow-light devices without quantum approaches, and has attracted enormous interest.^13^ The EIT-like effect generally arises from coupling between the bright and dark mode resonances, which results in an extremely sharp transparency window. The EIT-like effect markedly modifies the dispersive properties of the metamaterials, and can lead to slow-light phenomena. However, most metamaterials, whose unit cells are generally formed from metal, have relatively large ohmic loss,^14^ and the slow-light effects can only be tuned passively.^15,16^ All-dielectric metamaterials, whose unit cells are made of high-index semiconductor materials, such as silicon (Si) or germanium (Ge), offer a potential solution to the issue of metal loss.^17,18^ However, the EIT-like transparency window is very narrow.^19–21^

In this letter, we propose a hybrid metamaterial whose unit cell is composed of a silver (Ag) bar and a photoactive silicon (Si) ring. The structure combines the advantages of general metamaterials and all-dielectric metamaterials, such as low loss and high steep dispersion. By integrating photoactive Si into the functional unit cell of the hybrid metamaterial, a tunable EIT transparency window occurs under photoexcitation by a pump light, allowing for an all-optically tunable slow-light effect at a fixed frequency in the telecommunication window. This work represents an innovative approach for low loss, broadband, and all-optical actively tunable slow-light technology, and opens the possibility of designing novel chip-scale slow-light devices that could find utility in optical buffering.

## 2. Design and structure

**Fig. 1:**
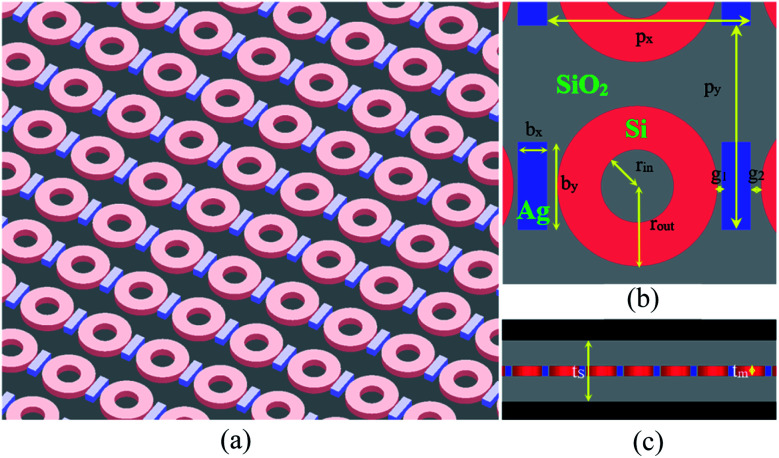
(a) Schematic of the hybrid metamaterial comprising a periodic array of Ag bars and Si rings embedded in a transparent SiO_2_ substrate. (b) Top view of the unit cell; the geometrical parameters are: *p*_*x*_ = 700 nm, *p*_*y*_ = 700 nm, *b*_*x*_ = 100 nm, *b*_*y*_ = 300 nm, *r*_out_ = 275 nm, *r*_in_ = 125 nm, *g*_1_ = 20 nm, and *g*_2_ = 30 nm. (c) Side view of the hybrid metamaterial; the geometrical parameters are: *t*_s_ = 1000 nm and *t*_m_ = 160 nm.

A schematic illustration of the proposed hybrid metamaterial is shown in Fig. 1. The structure comprises a periodic lattice made of a rectangular bar formed from Ag and a ring resonator formed from photoactive Si, and the bars and rings are embedded in a transparent silica (SiO_2_) substrate. The unit cells are arranged in both the *x* and *y* directions with periods of *p*_*x*_ = 700 nm, *p*_*y*_ = 700 nm, and the top view of one unit cell is shown in Fig. 1(b). The bar has a width of *b*_*x*_ = 100 nm and a length of *b*_*y*_ = 300 nm. The ring has an inside radius of *r*_in_ = 125 nm and an outside radius of *r*_out_ = 275 nm. The spacing between the bar and the adjacent ring is *g*_1_ = 20 nm and *g*_2_ = 30 nm. As the rings are placed asymmetrically on either side of the bar, the parameter *d* = *g*_2_ − *g*_1_ is used to track the difference between *g*_2_ and *g*_1_. As shown in Fig. 1(c), the thickness of the unit cells is *t*_m_ = 160 nm, and the thickness of the substrate is *t*_s_ = 1000 nm.

## 3. EIT-like effect response

**Fig. 2:**
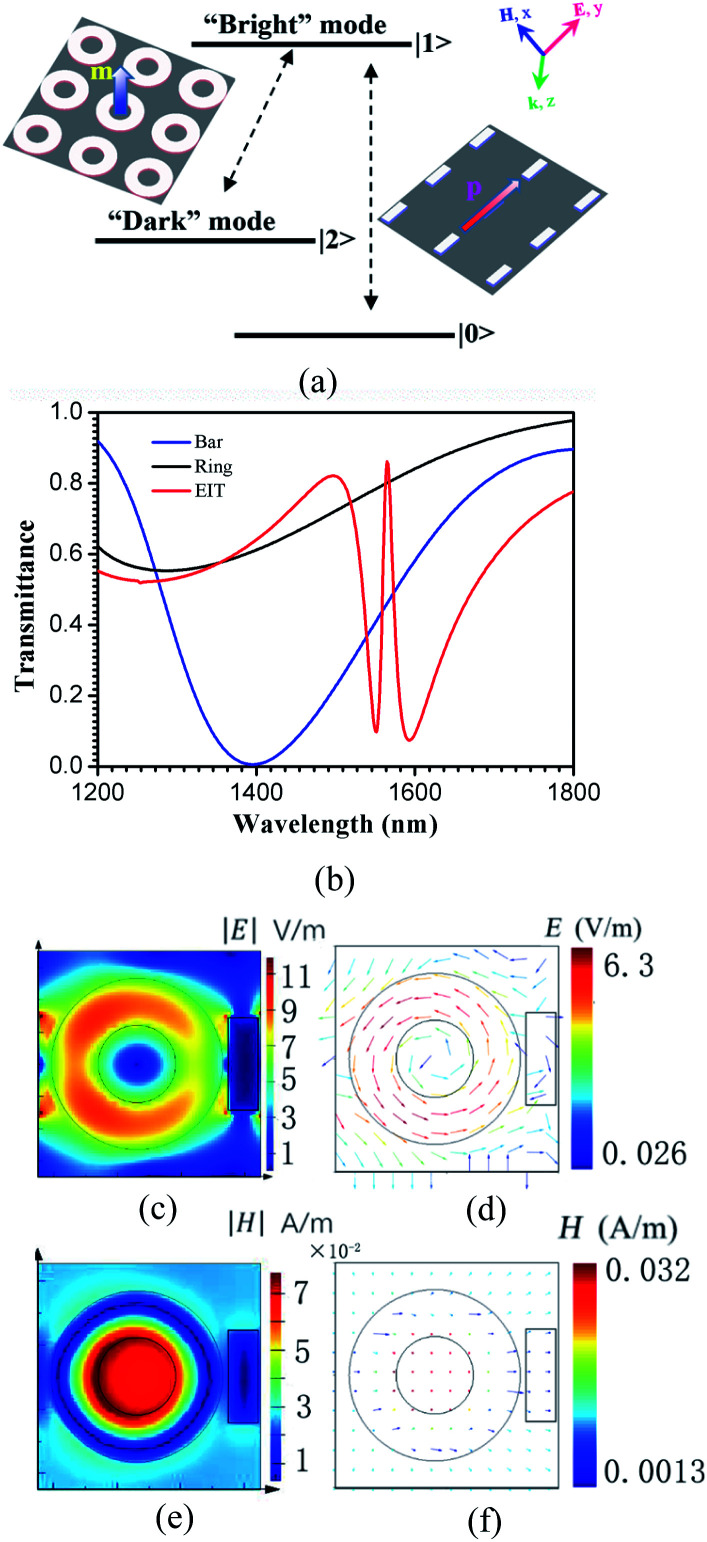
(a) Schematic of the interference between the bright and dark mode resonators. (b) Numerically simulated transmission spectra of the bars alone (blue line), rings alone (black line) and both together (red line), respectively. (c and d) simulated amplitudes and vector plots of the electric field at the EIT peak. (e and f) simulated amplitudes and vector plots of the magnetic field at the EIT peak.

In order to achieve an EIT-like effect in a metamaterial, unit cells consisting of two artificial resonant elements are generally used, namely, a radiative bright resonator that strongly couples with the light in free space and a dark resonator that weakly couples to the incident light. Due to the near-field interference between the two resonators, an EIT-like response will be achieved.^22^ In this letter, the unit cell of the designed hybrid metamaterial is composed of a metal (Ag) bar and a semiconductor (Si) ring. The bar serves as an electric dipole antenna, which couples strongly with the normal-incidence light and forms the “bright” mode resonance. The ring features a magnetic dipole mode that cannot be directly excited by light at normal incidence, as the magnetic field of the incident wave is perpendicular to the axis of the ring. However, it can couple to the bright mode through near-field coupling, which gives rise to the “dark” mode of the system. The collective bright and dark modes form a prototype three-level resonant system as shown in Fig. 2(a). An EIT-like effect can be generated by destructive interference between them.

To verify the characteristics of the designed hybrid metamaterial, we numerically calculated the transmittance spectra of the bars alone, the rings alone, and both together under normal-incidence light with the electric field parallel to the *y*-axis polarization and the magnetic field parallel to the *x*-axis. The results are shown in Fig. 2(b). The simulations were carried out using the finite-difference time-domain (FDTD) method with periodic boundary conditions, which is used to truncate the unit cell to mimic a 2D infinite structure in the *x*–*y* plane. Along the *z* direction, perfectly matched layers (PMLs) are used to absorb all the light coming out to the boundaries. To ensure accuracy, the length scale of the mesh was set as *d*_*x*_ = *d*_*y*_ = 20 nm and *d*_z_ = 10 nm, which are much smaller than *λ*_0_/10 throughout the simulation domain, where *λ*_0_ = 1500 nm is the central wavelength of the incident radiation. In the simulations, the Ag conductivity and SiO_2_ permittivity were represented by the experimental data from the Palik handbook.^23^ A conductive 3D material model was used to create the photoactive Si with a relative complex permittivity^23,24^ of *ε*_total_(*f*) = *ε* + *iσ*/2π*fε*_0_, where *ε* is the real permittivity, *σ* is the conductivity in units of S m^−1^, *ε*_0_ is the permittivity in a vacuum, and *f* is the incident light frequency. In these simulations, *ε* and *σ* are set as 13.69 and 0 S m^−1^, respectively, and *d* = *g*_2_ − *g*_1_ = 30 nm, 20 nm, or 10 nm. The other geometrical parameters were set to be identical to the values described in the caption of Fig. 1. The bar exhibits a bright resonance mode at 1395 nm, which is the basic and direct response to the incident plane wave. The bar possesses a broad resonance linewidth, shown by the blue line in Fig. 2(b). However, the ring does not directly respond to this excitation. When both the bar and ring are simultaneously arranged into the unit cell, a narrow transparency window, which is shown as the red line in Fig. 2(b), is clearly observed at a wavelength of 1565 nm because of the near-field coupling between the collective bright and dark modes. The electric and magnetic field profiles at the transmission peak are shown in Fig. 2(c) and (e), respectively. At the EIT peak, the energy in the array is concentrated in the collective dark mode with the magnetic dipoles in each of the rings as shown in Fig. 2(e). The vector plots of the electric fields are illustrated in Fig. 2(d). We found that the unique circular electric field, which is coupled from the bright modes, enhances the magnetic field in the center of the rings (as shown in Fig. 2(e)). As illustrated in Fig. 2(f), the direction of the magnetic field is perpendicular to the plane of the rings. In this configuration, the bars, which can be directly excited by the incident plane wave, act as a bright mode resonator, and the rings, which cannot be directly excited by this excitation, act as a dark mode resonator. The near-field coupling between them gives rise to an EIT-like effect.

**Fig. 3:**
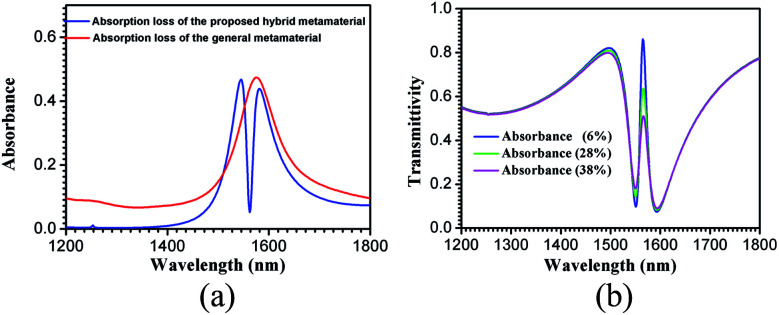
(a) Simulated absorption spectra (blue line) of the proposed hybrid metamaterial, and the absorption spectra (red line) of a metamaterial with same structure, except that the material (Si) of the ring is changed to silver (Ag). (b) EIT spectrum of the hybrid metamaterial with different absorbances: 6% (blue), 28% (green), and 38% (pink).

To illustrate the absorption loss of the hybrid metamaterial, its absorption spectrum (blue line) was simulated and is shown in Fig. 3(a). For comparison, the absorption spectrum (red line) of a metamaterial with the same structure, except that the material (Si) of the ring is changed to silver (Ag), is also shown in Fig. 3(a). A narrow region with very low absorption loss (<9%) is observed at the EIT-like spectrum peak of the hybrid metamaterial. However, the absorbance of the general metamaterial with the Ag unit cells is about 47% at the same wavelength. Thus, the absorption loss of the EIT-like hybrid metamaterial is much lower than that of the general metamaterial with the same structure. In addition, the EIT spectra of the hybrid metamaterial with different absorbances, namely, 6% (blue), 28% (green), and 38% (pink), were also simulated and are shown in Fig. 3(b). The full width at half maximum (FWHM) values of the spectra are broadened gradually with the enlargement of the absorbance.

## 4. Slow-light effect

**Fig. 4:**
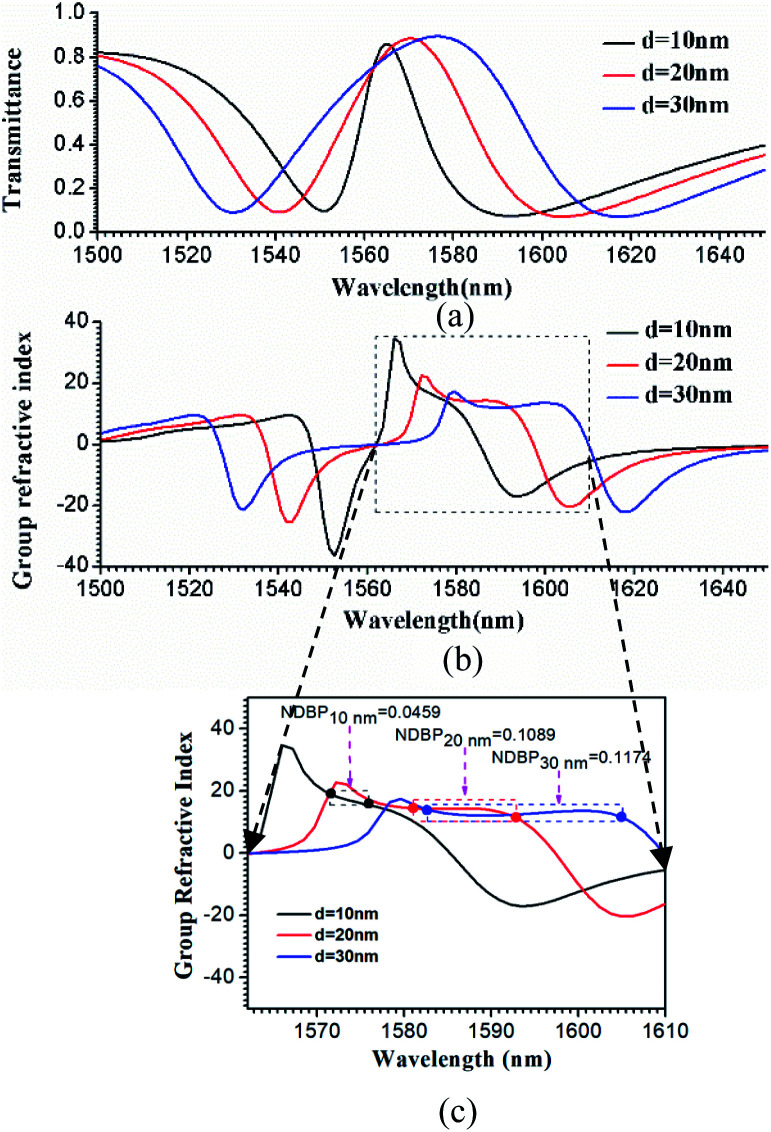
Calculated transmission spectra (a) and group refractive index (b) for the hybrid metamaterial with different *d* values of 10 nm, 20 nm, and 30 nm. (c) Magnification of the slow light region enclosed by the dotted black square in (b), in which the values of NDBP are marked.

The hybrid metamaterial has an EIT-like effect with low absorption loss and a steep dispersion, which is promising for realizing more efficient slow-light devices. The slow-light effect involves the reduction of the group velocity of the light waves propagating through the medium. The group index, *n*_g_, is the most direct and effective indicator to evaluate the slow light properties. The group index *n*_g_ can be calculated using the following equation:1
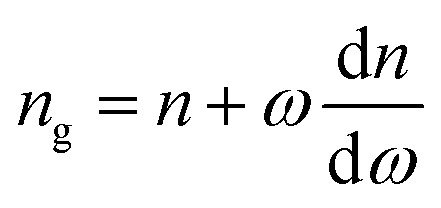
where *n* refers to the effective refractive index, and *ω* is the incident wave angle frequency. For the hybrid metamaterial, the effective refractive index *n* can be calculated from the scattering (*S*) matrix in the following form:^25^2
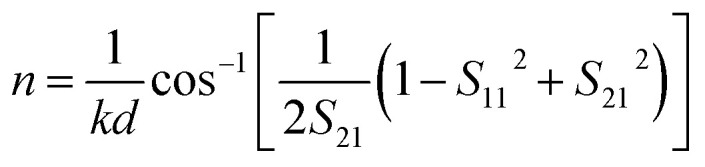
where *k* = 2π/*λ* is the wave vector of the incident light, *d* represents the thickness of the metamaterial, and *λ* is the optical wavelength. *S*_11_ = *E*_r_/*E*_i_ is the reflection coefficient, and *S*_21_ = *E*_t_/*E*_i_ is the transmission coefficient, where *E*_i_, *E*_r_, and *E*_t_ are the incident, reflected and transmitted electric field, and can be extracted from the FDTD simulation data. The EIT-like effect in the hybrid metamaterial was simulated using the FDTD method, and the transmission spectra are shown in Fig. 4(a) for *d* = 10 nm (black line), 20 nm (red line), and 30 nm (blue line). In the simulations, all other parameters were kept the same as in Fig. 1. The FWHMs of the EIT transparency windows were 16.15 nm, 32.29 nm, and 46.05 nm with peak transmittances of 85.6%, 88.4%, and 89.3% at wavelengths of 1564.95 nm, 1570.34 nm, and 1576.92 nm, respectively. Thus, it can be observed that the hybrid metamaterial has very high transmittance at the peaks of the EIT transparency windows.

Based on eqn (1) and (2), the group refractive index *n*_g_ were retrieved from the numerical data of the FDTD simulations and are plotted in Fig. 4(b). As shown in Fig. 4(b), with increasing *d*, the slow light region is gradually broadened. When *d* = 10 nm, 20 nm, and 30 nm, the average group indexes are *ñ*_g_ = 17.2, 14.7, and 12.5, and the slow-light bandwidths are 4.2 nm, 11.7 nm, and 22.2 nm. The bandwidth is normally determined as the wavelength range in which a group index variation of ±10% is tolerated.^26^ The most challenging issue in slow-light devices is simultaneously increasing the delay time and bandwidth. Particularly, for EIT-based slow light, a large delay usually appears in a narrow frequency band. Thus, an important parameter is introduced to evaluate the performance of slow light devices: the delay-bandwidth product (DBP = Δ*t* × Δ*f*, where Δ*t* is the time delay and Δ*f* is the bandwidth). DBP is important in optical communications and quantum optics, where a large DBP implies a large storage of data.^27^ In order to evaluate the capacity of slow light devices with different lengths, the expression for the DBP is modified to give the normalized DBP (NDBP), which is more useful when slow light devices have different lengths and different operating frequencies. The NDBP is defined as^28^3
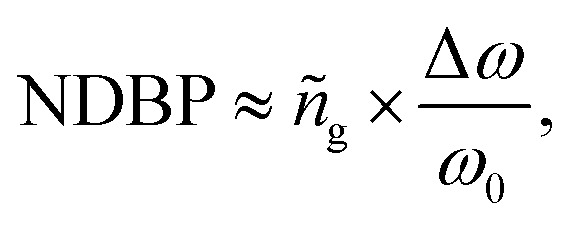
where Δ*ω*/*ω*_0_ is the normalized bandwidth of a slow light region and *ñ*_g_ is the average group index. In order to calculate the NDBP and present it more clearly, a magnification of the slow light region enclosed by the dotted black square in Fig. 4(b) is shown in Fig. 4(c). It can be seen that the value of NDBP in the hybrid metamaterial gradually increases from 0.0459 to 0.1174 with increasing *d*.

## 5. All-optical tunable slow-light effect

**Fig. 5:**
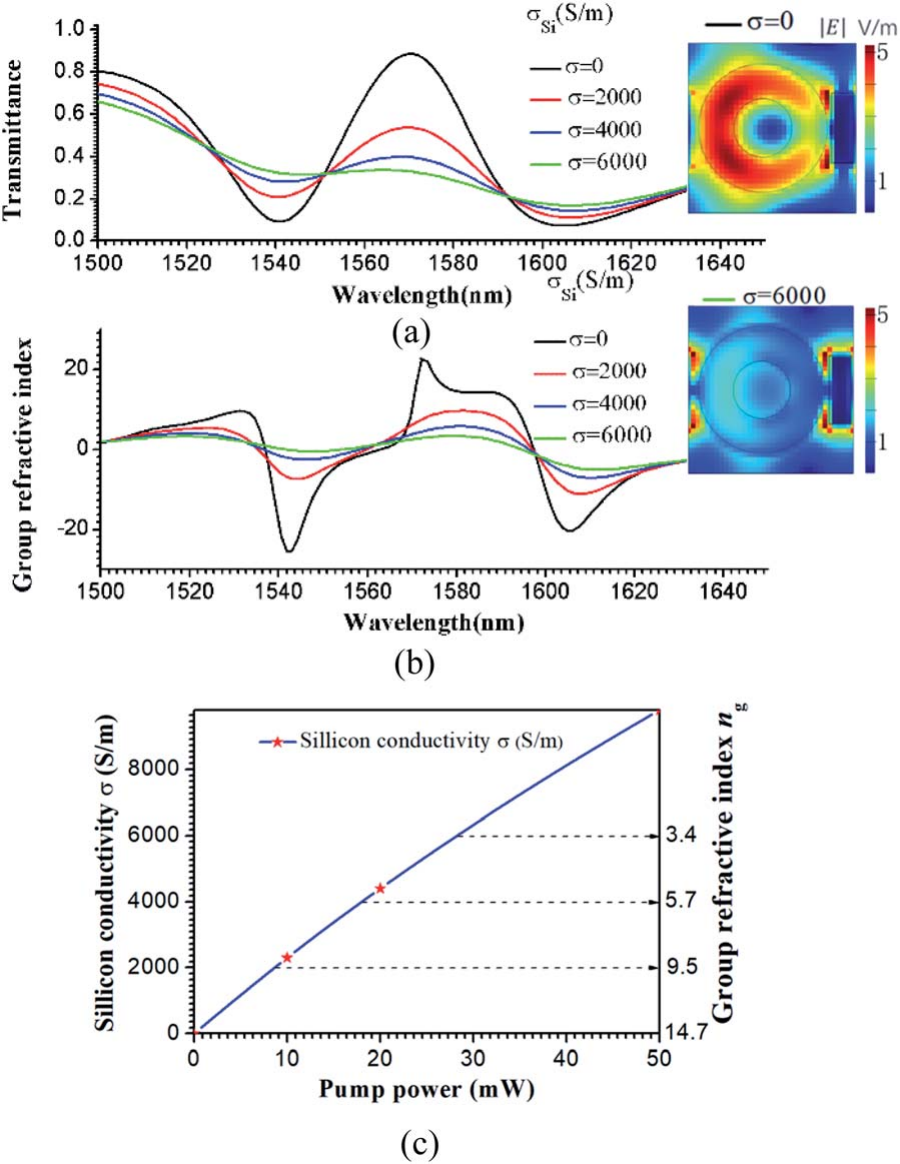
Simulated transmission amplitude (a) and group refractive index *n*_g_ (b) plotted as a function of the conductivity of Si; *σ* = 0 (black line), 2000 (blue line), 4000 (red line), and 6000 (green line) S m^−1^. The insets at the right side of (a) are the electric field amplitudes for *σ* = 0 (top), and 6000 (bottom) S m^−1^ (c) *ñ*_g_ and *σ* as a function of the laser power.

In some practical applications, it is highly desirable to achieve an all-optical controlled slow-light effect.^23^ Herein, we demonstrate active all-optical control of the slow light effect at a fixed frequency within the telecommunication window by integrating photoactive Si into the unit cell of the hybrid metamaterial. A pulsed laser with a central wavelength of 800 nm, which is typically utilized to excite electron–hole pairs in photoactive silicon, was collimated on the hybrid metamaterial array. The transmission properties of the hybrid metamaterial under different photoexcitations were investigated. On-to-off active control of the EIT-like resonance is demonstrated by changing the conductivity *σ* of Si and is shown in Fig. 5(a). The conductivity *σ* can be tuned by changing the laser power. Based on references ^12^ and 24, the conductivity *σ* as a function of the laser power is shown in Fig. 5(c). The stars represent experimental data from references ^12^ and 24, and the blue line is the fitting curve. In Fig. 5(a), it can be seen that when there is no photoexcitation, the conductivity *σ* = 0 S m^−1^, and an EIT peak is observed between two resonance dips with a transmission amplitude of 88.4% at 1570.34 nm. As the excitation power of the pump beam is gradually increased, the corresponding transmission undergoes a strong modulation. When the Si conductivity *σ* = 6000 S m^−1^, the peak of the transparency window gradually decreases to 32% and the EIT phenomenon vanishes. In the simulations, the other parameters were the same as in Fig. 1, except that *d* = 20 nm, and the Si conductivity *σ* = 0, 2000, 4000, or 6000 S m^−1^. Based on eqn (1) and (2), the group refractive index *n*_g_ depending on the optical wavelength was retrieved from the S matrix of the hybrid metamaterial for the various photoexcitation conditions and is shown in Fig. 5(b). The value of the average group index *ñ*_g_ could be actively tuned from 14.7 to 3.4. The *ñ*_g_ and the *σ* as a function of the laser power are shown in Fig. 5(c). An all-optical controlled slow-light effect can be achieved by tuning the pump laser power. The insets in Fig. 5 show that without the optical pump, the Si ring resonators are mainly dielectric and exhibit very small damping. The circular electric field is primarily focused on the Si ring array, as shown in the inset (top) in Fig. 5(a). The electric field in the bar is completely suppressed, which is characteristic of a typical EIT effect. Upon photoexcitation, the conductivity of the Si gradually increases, resulting in a redistribution of the electrical field in the structures. Furthermore, when *σ* = 6000 S m^−1^, the electric field in the ring array is completely suppressed, and the electric field distribution is localized at the Ag bar array. The hybrid metamaterial loses its slow light characteristic. Most significantly, the active tuning of the group refractive index demonstrated in this work may directly lead to optical controllable slow-light devices.

## 6. Conclusion

In summary, we proposed a hybrid metamaterial whose unit cell constitutes a bar formed of Ag and a ring formed of photoactive Si. This metamaterial combines the advantages of ordinary metamaterials and all-dielectric metamaterials, such as low loss and high steep dispersion. By incorporating the photoactive Si into the hybrid metamaterial unit cell, we numerically demonstrated an analogue of the EIT effect functioning within the telecommunication window and obtained all-optical control of the slow-light effect at a fixed frequency. The optically active tuning of the slow-light effect in the hybrid metamaterial may open avenues for designing active tunable optical buffering devices.

## Author contributions

Chengju Ma designed the structure of the hybrid metamaterial and the methodology and wrote the manuscript; Yuebin Zhang, Yao Zhang, and Shiqian Bao carried out the simulations; Jiasheng Jin and Mi Li created the data visualization; Dongming Li analyzed the simulation results; Yinggang Liu and Yiping Xu wrote the original draft.

## Conflicts of interest

There are no conflicts to declare.

## Supplementary Material
